# Evaluation on Potential Contributions of Protease Activated Receptors Related Mediators in Allergic Inflammation

**DOI:** 10.1155/2014/829068

**Published:** 2014-04-30

**Authors:** Huiyun Zhang, Xiaoning Zeng, Shaoheng He

**Affiliations:** ^1^Allergy and Clinical Immunology Research Centre, The First Affiliated Hospital of Liaoning Medical University, No. 2, Section 5, Renmin Street, Guta District, Jinzhou, Liaoning 121001, China; ^2^Department of Pathophysiology, Liaoning Medical University, Jinzhou, Liaoning 121001, China; ^3^Department of Respiratory Medicine, The First Affiliated Hospital of Nanjing Medical University, Nanjing 210029, China

## Abstract

Protease activated receptors (PARs) have been recognized as a distinctive four-member family of seven transmembrane G protein-coupled receptors (GPCRs) that can be cleaved by certain serine proteases. In recent years, there has been considerable interest in the role of PARs in allergic inflammation, the fundamental pathologic changes of allergy, but the potential roles of PARs in allergy remain obscure. Since many of these proteases are produced and actively involved in the pathologic process of inflammation including exudation of plasma components, inflammatory cell infiltration, and tissue damage and repair, PARs appear to make important contribution to allergy. The aim of the present review is to summarize the expression of PARs in inflammatory and structural cells, the influence of agonists or antagonists of PARs on cell behavior, and the involvement of PARs in allergic disorders, which will help us to better understand the roles of serine proteases and PARs in allergy.

## 1. Introduction

Protease activated receptors (PARs), a four-member family of GPCRs, can be cleaved by certain serine proteases within the extracellular amino terminus and expose a tethered ligand domain, which binds to and activates the receptors to initiate multiple signaling cascades. Therefore, these PAR-activating proteases are named as agonists of PARs. Since many of these proteases are produced during inflammation, PARs make important contributions to inflammatory tissue responses including exudation of plasma components, inflammatory cell infiltration, and tissue damage and repair in inflammation [1]. The PAR-activating serine proteases may derive from the circulation (e.g., coagulation factors), inflammatory cells (e.g., mast cell and neutrophil proteases), and multiple other sources (e.g., epithelial cells, neurons, bacteria, and fungi). Compounds that mimic or interfere with the PAR-activating processes are attractive therapeutic candidates: selective agonists of PARs may facilitate healing, repair, and protection, whereas protease inhibitors and PAR antagonists can impede exacerbated inflammation and pain.

In recent years, there has been considerable interest in the role of PARs [2, 3] in allergic inflammation, the fundamental pathologic changes in allergy. Since serine proteases have long been discovered to be actively involved in the pathologic process of inflammation and large amount of information on PARs is accumulated over the last two decades, it is necessary to write a literature review on PARs in allergy, which will help us to better understand the roles of serine proteases as agonists or antagonists of PARs in allergy.

## 2. Classification and Molecular Structures of PARs

Since the landmark study from Shaun Coughlin's group in which an expression cloning screen was used to identify the first human thrombin receptor known as PAR-1 [4], four numbers of this receptor class were found both in human and murine and designated as PAR-1, -2, -3, and -4, respectively [5]. As the newly found members of the typical seven trans-transmembrane GPCRs' family, the expression of PARs is found on the surface of cells from a wide variety of tissues [6].

The structure, activation mechanism, and signaling of PARs have been reviewed extensively [1, 5]. In brief, encoding genes for human PAR-1, -2, and -3 are located on chromosome 5 (q13), and for human PAR-4 the encoding gene is on chromosome 19 (p12). Although the location of PAR genes differs, high degree of structural similarity of all four genes predicts the conserved overall structure and function of these receptors [7, 8]. In both mouse and human, all four PARs have two exons: the first encoding a signal peptide and the second encoding the entire functional receptor protein [7]. Human PAR-1 protein is composed of 425 residues with 7 hydrophobic domains of a typical GPCR. The deduced sequence of human PAR-1 contains a potential cleavage site for thrombin within the amino tail: LDPR^41^↓S^42^FLLRN (where ↓ denotes cleavage) [4]. PAR-2 protein consists of 395 residues with the typical characteristics of a GPCR and with about 30% of the amino acid identity of human PAR-1. The extracellular amino acid terminus of 46 residues of PAR-2 contains a putative trypsin cleavage site, SKGR^34^↓S^35^SLIGKV [9]. PAR-2 is the most functionally distinct receptor in the PAR family as it is the only PAR which is not cleaved by thrombin. PAR-2 is most effectively cleaved by trypsin [9], tryptase [10], coagulation factors VIIa and Xa [11], the membrane type serine protease 1 (MT-SP1) [12], chitinase [13], and TMPRSS2, a type II transmembrane-bound serine protease [14]. Sharing about 28% sequence homology with human PAR-1 and PAR-2, human PAR-3 is activated in a very similar fashion to human PAR-1 with a thrombin cleavage site within the extracellular amino terminus LPIK^38^↓T^39^FRGAP [15]. Notably, mouse PAR-3 does not signal upon thrombin cleavage but functions instead via a unique cofactoring mechanism to support the activation of PAR-4 [16]. Human PAR-4, about 33% homologous to the other human PARs, is a 385-amino-acid protein with a potential cleavage site for thrombin and trypsin in the extracellular amino terminal domain PAPR^47^↓G^48^ YPGQV [17].

**Figure 1:**
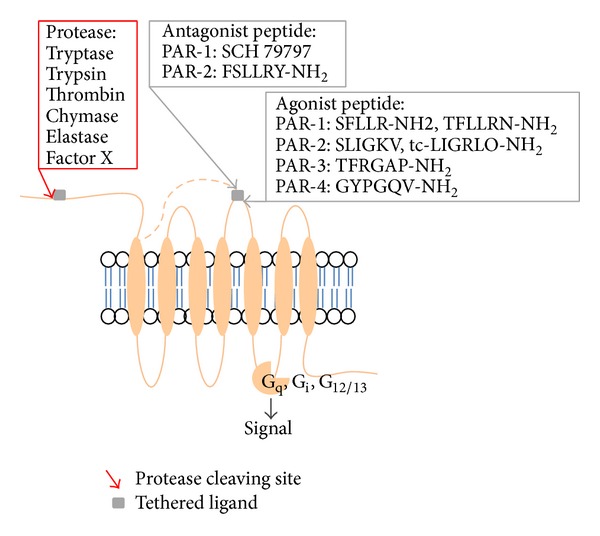
Activation mechanism of protease activated receptor (PAR). PARs are a group of four G protein-coupled receptors (GPCRs). Activation of PARs depends on the protease cleavage at the specific site of the extracellular N-terminal, upon which the exposed tethered ligand (gray square) binds to the second extracellular loop of PAR resulting in a series of cellular signaling events. The red arrow indicates cleavage site. The site for PAR-1 is at LDPR41↓S42FLLRN, PAR-2 is at SKGR34↓S35SLIGKV, PAR-3 is at LPIK38↓T39FRGAP, and PAR-4 is at PAPR47↓G48 YPGQV. PAR-2 antagonist peptide: FSLLRY-NH_2_; SCH 79797: a PAR-1 antagonist; the active peptides were PAR-1: SFLLR-NH_2_, TFLLRN-NH_2_; PAR-2: SLIGKV-NH_2_, transcinnamoyl- (tc-) LIGRLO-NH_2_; and PAR-3: TFRGAP-NH_2_ PAR-4, GYPGQV-NH_2_. S = Ser, F = Phe, L = Leu, R = Arg, T = Thr, I = Ile, G = Gl*y*, K = Lys, V = Val, O = Pyl, A = Ala, P = Pro, Y = Tyr, Q = Gln, and V = Val.

The novel activation mechanism distinguishes PARs from all other GPCRs though they share basic structural features. The general mechanism by which proteases cleave and activate PARs is similar: proteases cleave at specific sites within the extracellular amino terminus of the receptors; this cleavage exposes a new amino terminus, a cryptic N-terminal domain that serves as a “tethered ligand” domain, which binds to conserved region in the second extracellular loop of the cleaved receptor, and thereby activates the cleaved receptor. Synthetic peptides corresponding to the sequence of the “tethered ligand” are capable of activating the receptor independently of N-terminal proteolysis, confirming the self-activation model and providing a useful experimental tool for the specific activation of PARs [18] (Figure 1). These peptides include PAR-1 agonists SFLLR-NH_2_ and TFLLRN-NH_2_; PAR-2 agonists SLIGKV-NH_2_ and transcinnamoyl- (tc-) LIGRLO-NH_2_; PAR-3 agonist TFRGAP-NH_2_; and PAR-4 agonist GYPGQV-NH_2_. Activation of PARs results in a multiple cellular signaling events including cell shape, secretion, integrin activation, metabolic responses, transcriptional responses, and cell motility. PARs are “single use” receptors meaning that proteolytic activation is irreversible and the cleaved receptors are degraded in lysosomes [19].

## 3. Expression of PARs on Inflammatory Cells

Since inflammatory cells play a pivotal role in the pathogenesis of inflammation, what we should know first is the type and extent of PARs expression on inflammatory cells. As shown in Table 1, different PARs are expressed on mast cell, eosinophil, neutrophil, monocyte, macrophage, T cell, B cell, and dendritic cell (DC).

### 3.1. Expression of PARs on Mast Cells

It has been demonstrated that mast cells express all four PARs at both mRNA and protein levels. For example, PAR-1, -2, -3, and -4 are expressed on mouse mast cell line P815 cells [20–22] and MC/9 cells [20]. In addition, PAR-2 [23–25] and PAR-4 [24] are observed on the surface of human mast cell line HMC-1, and PAR-1 is found on murine bone marrow cultured mast cells [26]. The studies on human specimens show the presence of PAR-1 and PAR-2 in mast cells from various normal human tissues [27]; expression of PAR-2 and PAR-4 in mast cells both at protein and mRNA levels in the patients with postinfectious irritable bowel syndrome (PI-IBS) [28]; upregulated expression of PAR-2 in mast cells from ulcerative colitis tissues [25]; and the increased fraction of PAR-2-expressing mucosal mast cells in Crohn's specimens [23] (Table 1).

The expression of PARs appears to be regulated by cytokines. For example, interleukin- (IL-) 12 is able to downregulate PAR-2 expression [20], and IL-29 decreases PAR-1 expression [29] on P815 mast cells. On the other hand, granulocyte-macrophage colony-stimulating factor (GM-CSF) enhances expression of PAR-4 [30], tumor necrosis factor (TNF) [31] upregulates PAR-2 and PAR-4 expression, and RANTES increases expression of PAR-1 on P815 mast cells [32]. TNF also elevates PAR-2 expression on HMC-1 mast cells [23], and IL-12 upregulates PAR-4 expression on MC/9 mast cells [20]. Furthermore, tryptase-induced PAR-2 expression can be enhanced by IL-12 [20], TNF [31], RANTES [32], and IL-29 [29], and tryptase-induced PAR-3 and PAR-4 expression is upregulated by TNF on P815 mast cells [31]. It is also observed that trypsin-induced expression of PAR-1, -2, and -4 can be enhanced by RANTES [32] (Table 1).

### 3.2. Expression of PARs on Basophils

Little information on PAR expression on basophil is available. But a study showed lack of PAR expression on purified human basophils [33], and a report of the fact that trypsin rather than PAR-2 agonist induces histamine release from basophils [34] suggests that basophils may not express PARs.

### 3.3. Expression of PARs on Eosinophils

The expression of PAR-1 [35] and PAR-2 [35–37] has been observed on eosinophils. PAR-2 seems to be the major PAR receptor capable of modulating eosinophil functions [35]. It was reported that total numbers of eosinophils and the level of eosinophil expressing PAR-2 were significantly elevated in the nasal mucosa of seasonal allergic rhinitis (SAR) compared with the controls [37].

### 3.4. Expression of PARs on Neutrophils

Human peripheral blood neutrophils express PAR-1 and PAR-2, but not PAR-3 and PAR-4, proteins [38]. PAR-2 expression has also been observed on mouse pulmonary neutrophils [39].

### 3.5. Expression of PARs on Monocytes

The expression of PAR-1 [40–42], -2 [43–45], -3 [46], and -4 [47] on human monocytes is observed at both mRNA and protein levels. Interferon-gamma (IFN-*γ*) differentiated monocytes have increased expression of PAR-1 [42]. Monocyte surface PAR-2 expression is upregulated following static exposure to activated endothelial cell (EC) [44] (Table 1).

### 3.6. Expression of PARs on Macrophages

PAR-1, -2, and -3 expression has been showed in human macrophages [48, 49]. In Wistar rats, the expression of PAR-1, -2, -3, and -4 is revealed in airway macrophages (AMs) [50]. PAR-2 expression on human macrophages can be upregulated by dietary fatty acids (palmitic, stearic, and myristic) [51], but expression of PAR-1, -2, and -3 can be downregulated by IL-4 treatment [41]. Differentiation of human monocytes into macrophages by either macrophage colony-stimulating factor (M-CSF) or GM-CSF elicits enhanced expression of PAR-1, PAR-2, and PAR-3 [41]. There is a higher degree of PAR-1 protein staining in AMs from smokers compared with healthy controls (HC).

### 3.7. Expression of PARs on T Cells and B Cells

It has been showed that peripheral blood effector memory CD4(+) and CD8(+) T lymphocytes express PAR-1 [52] and that Jurkat T cells express PAR-2 [53]. In normal B cells, the expression of PAR-1 and PAR-3 is also reported [54].

### 3.8. Expression of PARs on DCs

It has been reported that monocyte-derived dendritic cells (MoDCs) do not express PARs [55, 56]. However, upon maturation with lipopolysaccharides (LPS), but not with TNF-*α* or CD40 ligand, DCs express PAR-1 and PAR-3 (not PAR-2 or PAR-4) [56]. IL-4 strongly downregulates PAR-1, -2, and -3 at both mRNA and protein levels in MoDCs [41]. Plasmacytoid DCs (pDCs) and myeloid DCs (mDCs) isolated from peripheral blood mononuclear cells (PBMC) express PAR-1 [55] and PAR-2 [57]. PAR-2 expression on mDCs is upregulated following German cockroach (GC) frass exposure [57].

## 4. Expression of PARs on Structural Cells

It has long been recognized that structural cells do have the ability to secrete proinflammatory mediators and cytokines, through which they actively participate in the pathogenesis of inflammation. More importantly, tissue remodeling processes in inflammation largely depend on structural cells and proteases. We therefore review the expression of PARs on epithelial cell, endothelial cell, fibroblast, smooth muscle cell (SMC), and keratinocyte in this section (Table 2).

### 4.1. Expression of PARs on Epithelial Cells

The A459 and BEAS-2B epithelial cell lines and primary human bronchial epithelial cells (HBECs) express PAR-1, -2, -3, and -4 as judged by RT-PCR and immunocytochemistry [58]. Both PAR-1 and PAR-2 are endogenously expressed in HBECs-16HBE14o-cells [59]. While PAR-1, -2, -3, and -4 expression is showed in alveolar epithelial cells of Wistar rats [50], PAR-2 appears to be a cellular receptor expressed prominently on epithelial cells of mice [60].

### 4.2. Expression of PARs on Endothelial Cells

Numerous studies have showed that PAR-2 is highly expressed on endothelial cells [50, 61, 62]. Stimulation with TNF-*α*, IL-1 *α*, and bacterial LPS elevates the expression of PAR-2 in a dose-dependent manner in cultured human umbilical vein endothelial cells (HUVECs) [63]. Macrophage migration inhibitory factor (MMIF) is found to enhance PAR-1 and PAR-2 mRNA expression in human endothelial cells [64], whereas phorbol ester treatment seems to decrease the expression of PAR-1 and PAR-2 [63]. In human endothelial cells, PAR-1 and *β*-arrestins form a preassembled complex [65]. Moreover, human cytomegalovirus (HCMV) induces expression of PAR-1 and PAR-3 but not PAR-4 proteins on HUVECs [66]. Since PAR-3 is postulated to represent a second thrombin receptor, its modest endothelial cell and platelet expression suggest that PAR-3 activation by alpha-thrombin is less relevant to physiological responses in these mature cells [67].

### 4.3. Expression of PARs on Fibroblasts

Human primary bronchial fibroblasts (HPBFs) express PAR-1, -2, and -3 but not PAR-4, and gingipains-induced secretion of hepatocyte growth factor (HGF) is significantly inhibited by RNA interference targeted at PAR-1 and PAR-2 [68, 69]. Similarly, both normal and fibrotic human lung fibroblasts express PAR-1, -2, and -3. There is no significant difference between normal and fibrotic fibroblasts in expression levels of PAR-1 and -3, whereas a fourfold higher expression level of PAR-2 is observed in fibrotic cells compared with normal cells [70]; in addition an important role of basic fibroblast growth factor (bFGF) in the regulation of functional PAR-2 expression in cultured RA synovial fibroblasts was reported [71]. It is reported that prostaglandin (PG) E2, via the prostanoid receptor EP2 and subsequent cAMP elevation, downregulates mRNA and protein levels of PAR-1 in human lung fibroblasts [72] and that PAR-2 upregulation by TNF-*α* may modulate myofibroblast proliferation [23].

### 4.4. Expression of PARs on SMCs

Human airway SMCs express PAR-1 and PAR-2 proteins [73, 74]. PAR-1, -3, and -4 are able to mediate thrombin-induced proliferation, migration, and matrix biosynthesis as well as generation of inflammatory and growth-promoting mediators in human vascular SMCs [75].

### 4.5. Keratinocytes

It is reported that TERT-2 cells constitutively express high levels of PAR-1 and PAR-2 and lower level of PAR-3 [76], whereas human keratinocytes express PAR-2 [11], which can be upregulated by MMIF [77].

## 5. Signal Transduction Pathways of PARs

**Figure 2:**
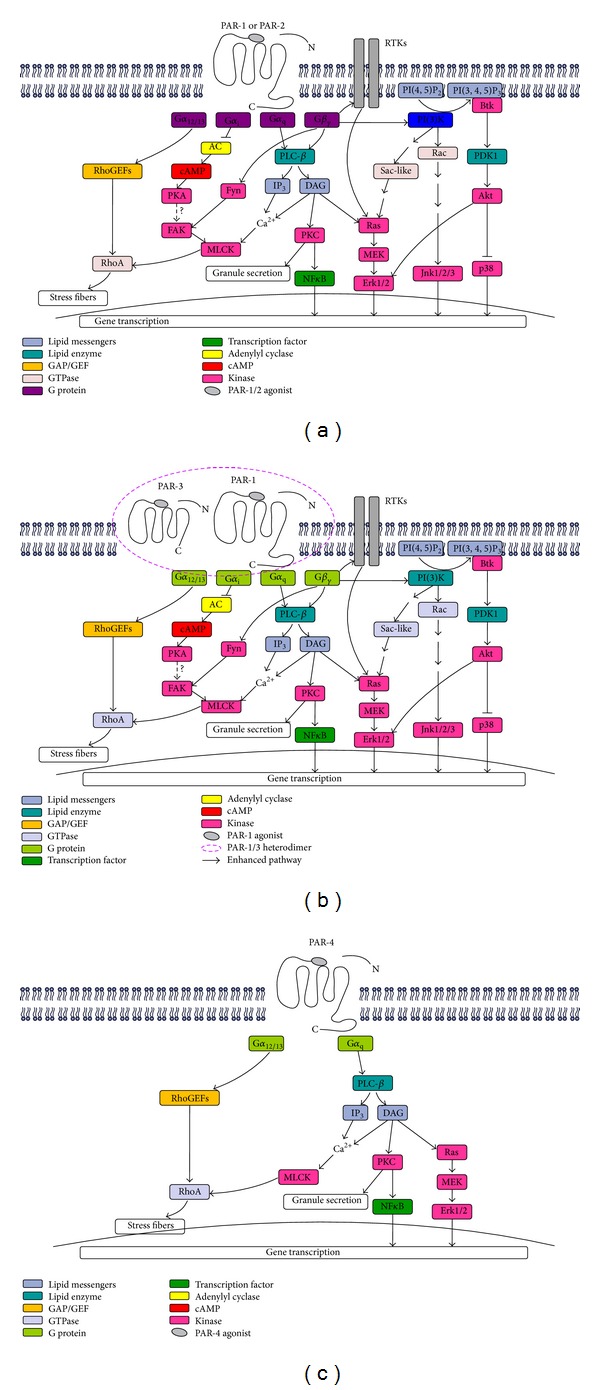
(a) Signal transduction pathways for PAR-1 and PAR-2. (b) Signal transduction pathways for PAR-1 and PAR-3. (c) Signal transduction pathways for PAR-4.

As shown in Figure 2, depending on the PAR subtype and the phenotype of PAR-expressed cell, the PAR family is able to stimulate a variety of intracellular signaling pathways. Like other “GPCRs,” the PARs signal via a variety of G proteins: both PAR-1 [78] and PAR-2 [79] through G*α*_q_, G*α*_i_, G*α*_12/13_, and G*β*_*γ*_; PAR-4 via G*α*_q_ and G*α*_12/13_ [78]; and PAR-3 via PAR-1 signaling by receptor dimerization. PAR-1 heterodimerization with PAR-3 alters the PAR-1/G*α*_13_ binding conformation, enhancing G*α*_13_ signaling. Heterodimerization does not likely affect PAR-1/  G*α*_q_ selectivity [80]. In addition, PAR-2 is able to signal via a non-G-protein mechanism that involves the beta-arrestin-mediated signaling [81]. Particularly in HMC-1 cell line, it was found that curcumin inhibits PAR-2 and PAR-4 mediated human mast cell activation, not by inhibition of trypsin activity but by the blocking of extracellular signal-regulated kinase (ERK) pathway [24], and thrombin mediates PAR-1 mediated mast cell adhesion through the activation of G(i) proteins, phosphoinositol 3-kinase, protein kinase C, and mitogen-activated protein kinase (MAPK) pathways [26].

Proteases mediating PAR-1 and PAR-2 activation differentially signal via MAPK cascades. In addition, the production of chemokines induced by PAR-1 and PAR-2 activation is suppressed by PI3K/Akt, thus keeping the innate immune responses of human oral keratinocytes in balance [82]. The CUX1 homeodomain protein is a downstream effector of PAR-2. Treatment of epithelial and fibroblastic cells with trypsin or the PAR-2 agonist peptide (AP) causes a rapid increase in CUX1 DNA binding activity. The stimulation of CUX1 was specific to PAR-2 because no effect was observed with thrombin or the PAR-1 AP. These results suggest a model whereby activation of PAR-2 triggers a signaling cascade that culminates with the stimulation of p110 CUX1 DNA binding and the transcriptional activation of target genes [83]. Thrombin induces RPE cell proliferation by joint activation of PLC-dependent and atypical PKC isoforms and the Ras-independent downstream stimulation of the Raf/MEK/ERK1/2 MAPK cascade [84].

## 6. Actions of Agonists and Antagonists of PARs in Inflammation

Since PARs are receptors expressed on various types of cells, their actions must be triggered or inhibited by agonists and antagonists of PARs. Hence, we review actions of agonists and antagonists of PARs in inflammation in the current section.

### 6.1. Influence of Agonists and Antagonists of PARs on Inflammatory Cell Migration

A significant increase in PAR-2 expression is observed on cell surface of neutrophils from septic patients as compared with HC. PAR-2 agonists (serine proteases as well as synthetic peptides) upregulate cell adhesion molecule expression and cytokine production and reduce transendothelial migration of neutrophils [85]. On the other hand, basolateral, but not apical, PAR-1 and PAR-2 activation with selective agonists decreases transepithelial resistance (TER) and thereby facilitates neutrophil transepithelial migration [86]. It is observed that activation of PAR-1 by thrombin stimulates directed migration of human eosinophils and thereby affects eosinophils in tissue and allergic inflammation [87].

### 6.2. Influence of Agonists and Antagonists of PARs on Cell Proliferation and Repair

Thrombomodulin (TM) acts as a thrombin receptor that modulates the duration of pERK nuclear retention and HUVEC proliferation in response to thrombin [88]. It has been found that thrombin and a PAR-1 APs stimulate proliferation of HPBFs [68] and that thrombin and FoxO factors functionally interact through PI3K/Akt-dependent FoxO phosphorylation leading to vascular SMC proliferation [89]. Furthermore, activated protein C (APC) acts through both PAR-1 and PAR-2 to activate Akt and to increase keratinocyte proliferation [90]. In mechanically wounded 16HBE 14o(-) epithelial cell layers in culture, PAR-1 and PAR-2 APs stimulate the rate of repair and enhance the formation of a fibrin provisional matrix to support the repair process. Locally expressed serine proteases of the coagulation cascade activate PAR-1 and PAR-2 to enhance fibrin formation and bronchial epithelial repair [91].

### 6.3. Influence of Agonists and Antagonists of PARs on Mediator and Cytokine Release from Cells

It has been observed that thrombin, tryptase, elastase, and trypsin, as well as APs of PAR-1, -2, and -4, induce IL-8 [92] and MCP-1 [93] release from A549 cells, suggesting that the actions of thrombin and trypsin may be via PAR-1 and -4, and cell responses to tryptase, trypsin, and elastase may be through PAR-2. Thrombin, trypsin, tryptase, elastase, SFLLR-NH_2_, and GYPGQV-NH_2_ stimulate also IL-6 release from monocytes [94]. A rank order of potency of the APs corresponding to the nascent N-termini of PAR-1, -2, and -4 appears as PAR-2 > PAR-4 > PAR-1 for induction of the release of IL-6 and IL-8 from A549, BEAS-2B, and HBECs. The APs of PAR-1, -2, and -4 also cause the release of PGE2 from A549 and HBECs [58]. Moreover, it is noticeable that thrombin, trypsin, tryptase, SFLLR-NH_2_, and SLIGKV-NH_2_ are capable of eliciting IL-6 release from T cells [95] (Table 3).

#### 6.3.1. Agonists of PAR-1

Thrombin is able to mediate induction of IL-1*β* and IL-6 cytokine production from PBMCs and PBMC cell proliferation in a PAR-1-dependent manner [47]. Thrombin can also induce IL-8 [96] and matrix metalloprotease- (MMP-) 9 release [97] from human primary dermal fibroblasts (HPDF) through activation of PAR-1. Similarly, thrombin and PAR-1 APs significantly stimulate vascular endothelial growth factor (VEGF) secretion from cultured human airway epithelial cells (HAEC) [98] and MMP-12 release from peritoneal macrophages [99]. A very recent study shows that MMP-1 causes activation of the nuclear factor-*κ*B (NF-*κ*B) pathway (p65/RelA) in endothelial cells, and this response is dependent upon activation of  PAR-1 [100] (Table 3).

#### 6.3.2. Agonists of PAR-2

In the alveolar macrophage cell lines (MH-S cells) and peritoneal macrophage cell lines (RAW264.7 cells), GC extract activates PAR-2 and thereby produces TNF-*α*. GC extract can also enhance TNF-*α* production by alveolar macrophages through the PAR-2 pathway [49]. The observation that trypsin and SLIGKV-NH_2_ are able to stimulate an increase in vascular cell adhesion molecule- (VCAM-) 1 expression and the release of IL-8 and granulocyte colony-stimulating factor (G-CSF) from bronchial fibroblasts suggests the importance of PAR-2 in promoting neutrophilic airway inflammation [68]. A finding demonstrates that neutrophil elastase can increase PAR-2 expression and MUC5AC mucin release [101] may also implicate the involvement of PAR-2 in airway inflammation. While treatment with SLIGKV-NH_2_ at the apical or basolateral cell surface of epithelial cells induces GM-CSF, ICAM-1, TNF-*α*, MMP-1, and MMP-10 secretion [102], PAR-2 agonist also provokes the production of thymic stromal lymphopoietin (TSLP) in the skin of mice [103]. Since these mediators and cytokines are important promoters of inflammation, PAR-2 should be a pivotal contributor of inflammation. Moreover, it is reported that mite-derived serine protease activity may contribute to the pathogenesis of atopic dermatitis (AD) by activating keratinocytes via PAR-2 activation [104]. Allergen-induced, PAR-2/epidermal growth factor receptor- (EGFR-) mediated signaling may also decrease epithelial resistance and promotes junction disassembly [105], thereby promoting epithelial inflammation.

It has been showed that tryptase, trypsin, SLIGKV-NH_2_, tc-LIGRLO-NH_2_, but not thrombin, elastase, and SFLLR-NH_2_ can induce IL-8 and lactoferrin secretion from peripheral blood neutrophils [38], implicating that the actions of tryptase and trypsin are likely via the activation of PAR-2. Studies on mice show that neutrophils from antiphospholipid (aPL) antibody-treated mice express PAR-2 and that stimulation of this receptor leads to neutrophil activation [106]. Antineutrophil cytoplasmic antibodies against proteinase 3 (PR3) activate human monocytic THP-1 cells in a PR3- and PAR-2-dependent manner [45].

Human eosinophils express PAR-1 and -2, and PAR-2 is the major PAR receptor that is capable of modulating eosinophil function. Trypsin and the PAR-2 APs are seen in triggering shape change, release of cysteinyl leukotrienes, and, most obviously, generation of reactive oxygen species in eosinophils [35]. A study demonstrates that a specific tethered peptide ligand for PAR-2 potently induces superoxide production and degranulation may support the finding above [36]. It is reported that SLIGKV-NH_2_ and tc-LIGRLO provoke histamine release from skin mast cells and that trypsin is able to induce a bell shape increase in tryptase release from tonsil mast cells [107]. In HUVEC, trypsin-induced IL-8 release is inhibited by the inhibitor peptide of PAR-2 Phe-Ser-Leu-Leu-Arg-Tyr-NH_2_ (FSLLRY-NH_2_), suggesting that the action of trypsin on HUVEC is most likely through activation of PAR-2 [108]. Very recently, it is reported that PAR-3 cooperates with PAR-1 to mediate the effect of thrombin on cytokine production and VCAM-1 expression in endothelial cells, but ICAM-1 expression in endothelial cells requires PAR-3 without PAR-1 [54]. It has been reported that aspartate proteases from* Alternaria* induce GM-CSF, IL-6, and IL-8 production and calcium response in airway epithelium through PAR-2 [109] and that* Alternaria*-derived aspartate proteases cleave PAR-2 to activate eosinophil degranulation [110], which may add another novel mechanism for activation of PARs in the development and exacerbation of airway allergic diseases (Table 3). We have recently found that tryptase can induce the release of IL-6 and TNF-*α* from astrocytes via PAR-2-MAPKs or PAR-2-PI3K/Akt signaling pathway, which reveals PAR-2 as a new target actively participating in the regulation of astrocytic functions [111].

#### 6.3.3. Antagonists of PARs

Potent heterocycle-based peptide-mimetic antagonists of PAR-1, RWJ-56110 [112], and RWJ-58259 [113] are potent, selective PAR-1 antagonists, which bind to PAR-1, interfere with calcium mobilization and cellular functions (platelet aggregation and cell proliferation), and do not affect PAR-2, -3, or -4. Both PAR-1 and PAR-4 activation peptides were significantly inhibited by affinity-purified anti-PAR-1-IgY and anti-PAR-4-IgY and by the specific PAR-1 antagonist BMS 200261 [114]. A cell-penetrating pepducin antagonist of PAR-4 (P4pal-10) dose-dependently diminishes the severity of endotoxemia and preserves liver, kidney, and lung function of mice, suggesting that inhibition of PAR-4 signaling in neutrophils could be protective in systemic inflammation and DIC [115]. Using a fluorescence assay, a novel compound, GB88, is shown to antagonize PAR-2-induced intracellular Ca(2+) release in human monocyte-derived macrophages, being 1000 times more potent than a novel small molecule PAR-2 antagonist N1-3-methylbutyryl-N4-6-aminohexanoyl-piperazine (ENMD-1068). GB88 inhibits also the acute paw edema induced in Wistar rats by PAR-2 agonist 2-furoyl-LIGRLO-NH2 or mast cell *β*-tryptase, without inhibiting proteolytic activity of tryptase* in vitro* [116, 117].

## 7. Roles of PARs in Allergic Diseases

Accumulated evidence suggests that PARs are closely related to allergic inflammation, but the detailed relationship between PARs and allergic diseases remains obscure. We therefore review the known roles of PARs in allergic diseases in the current section (Table 4).

### 7.1. Roles of PAR-1 in Allergic Diseases

#### 7.1.1. In Allergic Rhinitis (AR)

It has been reported that thrombin is increased in nasal secretion of the patients with chronic rhinosinusitis and thrombin and PAR-1 APs stimulate VEGF secretion from cultured HAEC [98], suggesting that thrombin may play a role in nasal polyp formation by stimulating VEGF production from airway epithelial cells.

#### 7.1.2. In Asthma

Heterozygous PAR-1 mice have less allergic inflammation but PAR-1 agonist worsens it. Allergic bronchial inflammation is worsened in mice that receive adoptive transfer of PAR-1 agonist-treated Th2 cells compared to controls. Low concentrations of thrombin suppress but high dose of it enhances maturation and secretion of cytokines in DCs [118]. The expression of PAR-1 is upregulated by thrombin that induces the expression of TGF-*β*1 to promote airway remodeling in OVA-allergic rats [119]. An integrated effect is also observed in one haplotype cluster consisting of both regions of the EGFR gene and the PAR-1 gene, suggesting the possibility that the integrated effect of functionally related EGFR and PAR-1 genes (haplotype cluster) is associated with susceptibility to airway hyperresponsiveness (AHR) [120].

### 7.2. Roles of PAR-2 in Allergic Diseases

#### 7.2.1. In AR

The expression levels of PAR-2 mRNA and immunoreactivity for PAR-2 in the nasal mucosa of AR are significantly upregulated as compared with normal nasal mucosa [121], suggesting that PAR-2 is very likely to be involved in allergic nasal inflammation. Indeed, it is found that house-dust mite (HDM) induces a higher secretion rate and numbers of responding glands in the AR group of patients than in the control group. Since PAR-2 is highly expressed in nasal mucosa, PAR-2 activating peptide provokes similar responses in nasal mucosa, and both HDM and PAR-2 activating peptide induced responses are suppressed by ENMD-1068; the involvement of PAR-2 in AR is confirmed [122]. In addition, HDM-induced PAR-2 activation and fluid secretion in porcine airway mucosa suggest a role for PAR-2 in mucociliary clearance and fluid hypersecretion of airway mucosa [123]. It is found that suppression of connexin 26 in HDM-sensitized AR patients is related to a PAR-2 mediated pathway and may be involved in the initiation and maintenance of AR [124]. Stimulation of elevated IL-6 and IL-8 production in primarily cultured nasal epithelial cells (NECs) by a major allergen of HDM Der p1 may also contribute to AR. Der p1 induced IL-6 and IL-8 production which seems to be associated with the PAR/PI3 K/NF*κ*B signaling pathway [125].

Furthermore, the abundant presence of PAR-2 in different cell types including eosinophils and epithelial cells in the nasal mucosa suggests that PAR-2 may contribute to the pathogenesis of seasonal AR [37]. Based on the colocalization of PAR-2 and tachykinins in trigeminal sensory neurons innervating the nasal mucosa, it is suggested that, following an activation of PAR-2 in tachykinergic neurons by trypsin and mast cell tryptase, there may be a triggering of tachykinin-mediated neurogenic inflammation in allergic or nonallergic rhinitis [126].

#### 7.2.2. In Asthma

It is observed that, in normal and asthmatic subjects, epithelial staining intensity of PAR-1 and PAR-3 is greater than that of PAR-4. However, PAR-2 staining in asthmatic epithelium is increased in comparison with normal epithelium [127], which suggests that PAR-2 may be involved in the pathogenesis of asthma. In fact, activation of PAR-2 has been found to induce contraction of human airways and potentiate contraction to histamine and therefore may contribute to asthma [128]. Through a PAR-2-dependent mechanism, chitinase 3-like 1 protein promotes bronchial SMC proliferation and migration [129]. Basolateral PAR-2 activation in the mouse airways leads to increased anion secretion through apical calcium-activated chloride channels, which is more pronounced in allergic animals [130]. Compared with wild-type animals, eosinophil infiltration is inhibited by 73% in mice lacking PAR-2 and increased by 88% in mice overexpressing PAR-2. Similarly, airway hyperreactivity to inhaled methacholine is diminished by 38% in mice lacking PAR-2 and increased by 52% in mice overexpressing PAR-2. PAR-2 deletion also reduces IgE levels to OVA sensitization. These results indicate that PAR-2 contributes to the development of immunity and to allergic inflammation of the airway [131].

HDM exposure to wild-type (Wt) mice causes a profound influx of eosinophils in bronchoalveolar lavage fluid (BALF) and accumulation of eosinophils in lung tissue. Both of them are strongly reduced in PAR-2 KO mice. PAR-2 KO mice demonstrate also attenuated lung pathology and protein leak in the bronchoalveolar space, accompanied by lower BALF levels of the anaphylatoxins C3a and C5a [132]. Through its serine protease activity, HDM potentiates capsaicin-evoked Ca(2+) transient in mouse pulmonary sensory neurons via PAR-2-phosholipase C-protein kinase C intracellular transduction cascade [133]. HDM extract also upregulates calreticulin (CRT) protein, activates PAR-2, increases IL-6 expression, and induces the proliferation of asthmatic bronchial SMC [134].

GC fecal remnants contain active serine proteases which augment TNF-*α*-induced MMP-9 expression by a mechanism involving PAR-2, ERK, and AP-1 [135]. GC extract has a direct effect on HAEC, in particular generating [Ca(2+)](i) oscillations through activation of PAR-2 [136]. PAR-2-deficient mice have significantly reduced AHR, Th2, and Th17 cytokine release, serum IgE levels, and cellular infiltration compared to Wt mice when sensitizaed to GC frass [137].* Alternaria* proteases act through PAR-2 to induce rapid increases in human airway epithelial [Ca(2+)](i)* in vitro* and cell recruitment* in vivo*, the critical early steps in the development of allergic asthma [138]. Through the activation of PAR-2, allergen-derived proteases are sufficient to induce CCL20 (chemokine C-C motif ligand 20) and GM-CSF production in the airways leads to increased recruitment and/or differentiation of mDC populations in the lungs, which likely plays an important role in the initiation of allergic airway responses [139].

A report that tryptase and SLIGKV-NH2 act on airway smooth muscle and lead to homologous beta-adrenergic desensitization via PAR-2 activation [140] suggests a relatively novel mechanism involved in asthma. Supernatants of human SMCs treated with the major mast cell product tryptase have increased chemotactic activity for the HMC-1 mast cell line. The effect depends on an intact catalytic site for tryptase and can be induced by a peptide agonist for PAR-2, indicating that the action of tryptase is via PAR-2 activation [141]. Moreover, PAR-2 activation, by sensitizing the transient receptor potential vanilloid 1 (TRPV1) in primary sensory neurons, may play a role in the exaggerated cough observed in certain airways inflammatory diseases such as asthma and chronic obstructive pulmonary disease [142]. Desensitization of PAR-2 by repetitive agonist stimulation or siRNA-mediated PAR-2 knockdown reveals that chitinase-mediated [Ca(2+)](i) increase is exclusively mediated by PAR-2 activation. Chitinase is found to cleave the cleavage site of PAR-2 and enhance IL-8 production, indicating that exogenous chitinase is a potent proteolytic activator of PAR-2 in human airway epithelial cells [13]. In contrast, SLIGRL-NH_2_ has been found to inhibit the development of airway eosinophilia and hyperresponsiveness in allergic mice through COX-2-mediated generation of the anti-inflammatory mediator PGE2. SLIGRL also displays bronchodilator activity in allergic mice. These studies support the concept that PAR-2 exerts predominantly bronchoprotective actions within allergic murine airways [143]. It is also found that PAR-2 AP pretreatment is able to inhibit airway hyperresponsiveness and bronchoconstriction and to modulate the immune response induced by allergic challenge in sensitized rabbits [144].

#### 7.2.3. In Allergic Skin Disorders

It has been reported that certain proteases signal to cells by activating PARs in the skin. Recent studies have revealed aberrant expression and activation of serine proteases and PAR-2 in the lesional skin of AD patients. Upregulated proteases stimulate PAR-2 and lead to the production of cytokines and chemokines involved in inflammation and immune responses, itching sensation, and sustained epidermal barrier perturbation with easier allergen penetration. In addition, PAR-2 is an important sensor for exogenous danger molecules, such as exogenous proteases from various allergens, and plays an important role in AD pathogenesis [145]. Defective skin barrier facilitates allergen and microbe penetration and generates danger signals leading to PAR-2 activation, which triggers the production of the major pro-Th2 cytokine TSLP and TNF-*α* [146]. It is also found that allergens with protease activity can influence the epidermal permeability barrier homeostasis through PAR-2 activation and consequent modulation of the calcium ions in skin [147]. PAR-2 may be involved in passive cutaneous anaphylaxis-induced scratching behavior in ICR mice [148]. Intradermal injections of histamine and SLIGRL-NH_2_ induce scratching in naive mice, but protease-associated itch and allergy-associated itch are different from those of histamine-induced itch [149]. It is reported that PAR-2 may also play a crucial role in type IV allergic dermatitis [150].

#### 7.2.4. In Allergic Colitis

Increased levels of serine proteases activating PAR-2 are found in the lumen and colonic tissue of inflammatory bowel diseases (IBD) patients. PAR-2 activity and proinflammatory cytokines impair epithelial barrier, facilitating the uptake of luminal aggressors that perpetuate inflammation in IBD [151]. Trypsin and tryptase expression and release are increased in colonic biopsies from irritable bowel syndrome (IBS) patients compared with control subjects. Biopsies from IBS patients release mediators that sensitize murine sensory PAR-2 expressing neurons in culture. Supernatants from colonic biopsies of IBS patients also cause somatic and visceral hyperalgesia and allodynia in mice. These pronociceptive effects are absent in PAR-2-deficient mice, indicating that proteases released in IBS can directly stimulate sensory neurons and generate hypersensitivity symptoms through the activation of PAR-2 [152]. PAR-2 mediated relaxation system in colonic smooth muscle is suppressed in experimental colitis rat model, which may contribute to motility disorders in IBD [153].

Activation of PAR-2 on epithelial cells may directly affect cytoskeleton contraction by triggering phosphorylation of myosin light chain with subsequent changes in tight junction permeability [154]. Dexamethasone treatment improves PAR-2 agonist-induced visceral hypersensitivity but does not prevent PAR-2 agonist-induced increase in colonic permeability in rats [155]. It has been showed that PAR-2 is upregulated on ileal mucosal mast cells in Crohn's ileitis, which may contribute to perpetuating the inflammatory process in the intestinal mucosa in Crohn's ileitis [23].

It is reported that GB88 is a potent antagonist of PAR-2 activation in colonocytes. Acute colonic inflammation induced in rats by SLIGRL-NH_2_ is inhibited by oral administration of GB88 with markedly reduced edema, mucin depletion, PAR-2 receptor internalization, and mastocytosis. Chronic trinitrobenzenesulfonic acid-induced colitis in rats is ameliorated by GB88, which reduces mortality and pathology (including colon obstruction, ulceration, wall thickness, and myeloperoxidase release) more effectively than the clinically used drug sulfasalazine, suggesting the therapeutic potential for PAR-2 antagonist in inflammatory diseases of colon [116].

### 7.3. Roles of PAR-4 in Allergic Diseases

#### 7.3.1. In Allergic Colitis

PAR-4 is functionally expressed in rat colon and its activation induces contraction of the longitudinal muscle both through tetrodotoxin sensitive release of acetylcholine and release of tachykinins, probably from sensory nerves [156]. PAR-4 agonists modulate colonic nociceptive response and inhibit colonic hypersensitivity and primary afferent responses to pronociceptive mediators. Endogenous activation of PAR-4 also plays a major role in controlling visceral pain. These results identify PAR-4 as a previously unknown modulator of visceral nociception [157]. The PAR-4 activation is endogenously involved as a feedback loop to attenuate inflammatory colonic hyperalgesia to colorectal distension [158]. Ulcerative colitis (UC) fecal supernatants, cathepsin G (Cat-G), and PAR-4 agonist can increase both paracellular permeability and myeloperoxidase activity [159]. Increased colonic paracellular permeability that is triggered by UC fecal supernatants can be blocked by both specific Cat-G inhibitor (SCGI) (77%) and pepducin P4pal-10 (PAR-4 antagonist) (85%) [160]. UC supernatant also promotes colonic hyposensitivity to distension, an effect can be mimicked by PAR-4 AP or Cat-G. Blockage of PAR-4 or Cat-G inhibition results in colonic hypersensitivity [161].

## 8. Summary: Potential Roles of Agonists and Antagonists of PARs in Allergic Inflammation

**Figure 3:**
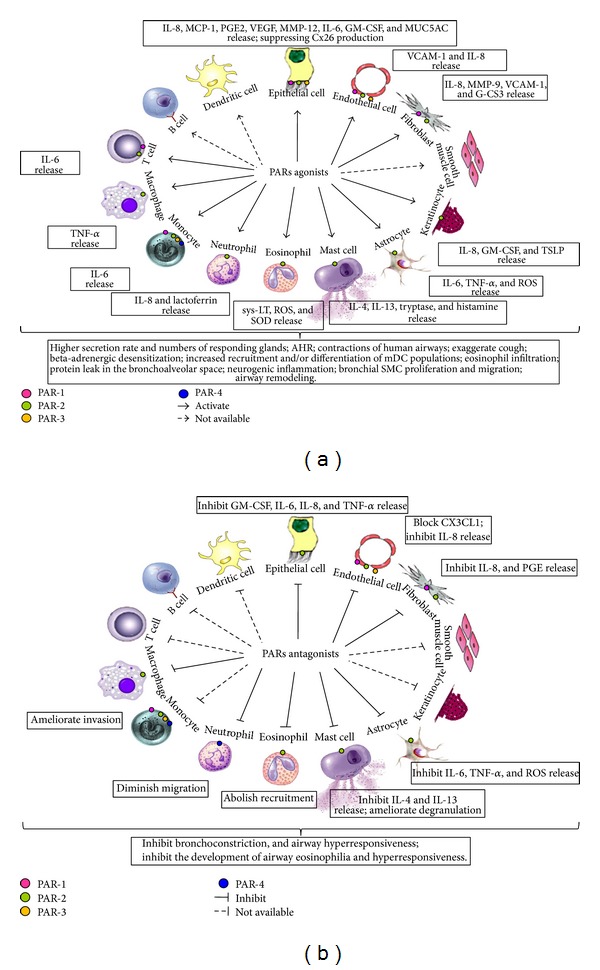
(a) Potential roles of agonists of PARs in allergic inflammation. (b) Potential roles of antagonists of PARs in allergic inflammation.

The potential roles of agonists (Figure 3(a)) and antagonists of PARs ( Figure 3(b)) in allergic inflammation are summarized. Although PARs as a unique family of GPCRs are widely expressed on inflammatory cells, each member of the family appears to selectively be expressed on different cell types. For example, mast cells express PAR-1, -2, -3, and -4 under different conditions, but eosinophils and neutrophils seem to only express PAR-1 and PAR-2. Selective expression of subtypes of PARs has also been found in different structural cells such as epithelial cells, SMCs, and fibroblasts.

Agonists of PARs including proteases can modulate neutrophil transepithelial migration, provoke proliferation of HUVEC, HPBFs, vascular SMC, and keratinocyte, and induce murine wound healing, fibrin formation, and bronchial epithelial repair. They are also able to induce release of various types of cytokines and proinflammatory mediators from inflammatory cells. In addition, increased vascular leakage and enhanced bronchial smooth muscle contraction can be elicited by agonists of PARs.

On the other hand, selective PAR-1 antagonists interfere with platelet aggregation and cell proliferation, whereas an antagonist of PAR-4 diminishes the systemic inflammation and local neutrophilic inflammatory responses. A novel PAR-2 antagonist GB88 inhibits the acute paw edema induced by PAR-2 agonist or mast cell *β*-tryptase.

In AR, since PAR-2 is highly expressed in nasal mucosa, PAR-2 activating peptide provokes higher secretion rate and numbers of responding glands in nasal mucosa, and both HDM- and PAR-2 activating peptide induced responses are suppressed by ENMD-1068; the involvement of PAR-2 in AR is confirmed. In asthma, PAR-2 staining in asthmatic epithelium is increased and activation of PAR-2 has been found to induce contraction of human airways and potentiate contraction to histamine, and therefore may contribute to asthma. Studies that revealed aberrant expression and activation of serine proteases and PAR-2 in the lesional skin of AD patients, increased levels of PAR-2 in the lumen and colonic tissue of IBD patients, and upregulated PAR-2 on ileal mucosal mast cells in Crohn's ileitis implicate that PAR-2 is likely involved in the pathogenesis of these diseases. Moreover, inhibition of acute colonic inflammation by GB88 suggests the therapeutic potential for PAR-2 antagonist in inflammatory diseases.

In conclusion, increased expression and activation of PARs, particularly PAR-2, are closely associated with inflammatory conditions, suggesting that this relatively novel receptor family is likely to contribute to inflammatory process and subsequently facilitates allergic disorders. However, lack of clinically effective anti-PAR drugs and multihospital involved clinical investigation is not supportive for the statement that PARs play key roles in allergy as yet.

## 9. Future Work

In order to further evaluate the potential roles of PARs in allergy, the following work should be done: (1) investigating the reasons for selective expression of PARs on different cell types; (2) investigating influence of different types of allergens on expression and functions of PARs; (3) investigating further the effects of various inflammatory mediators on expression and functions of PARs, and vice versa; (4) investigating PARs in allergic conditions in a more and better designed clinical way; (5) developing clinically effective drugs for treatment of allergy.

## Figures and Tables

**Table 1:** Expression of protease activated receptors (PARs) on inflammatory cells.

Cell type	Expression of PARs				Regulator of PARs	
	PAR-1	PAR-2	PAR-3	PAR-4	Upregulation	Downregulation
MC	+ [22, 27]	+ [22, 23, 25, 27, 28]	+ [22]	+ [22, 28]	RANTES for PAR-1 [32]; TNF-*α* for PAR-2 [23, 31] and PAR-4 [31]; IL-12 for PAR-4 [20]; GM-CSF for PAR-4 [30]; AC allergen rPer a1.01 for PAR-1,2,4 [22].	IL-29 for PAR-1 [29]; IL-12 for PAR-2 [20].

Eos	+ [35, 36]	+ [35–37]	+ [36]	− [36]	na	na

Neu	+ [38]	+ [38, 39, 106]	− [38]	− [38]	na	Simvastatin and pravastatin for PAR-2 [106]; GC for PAR-2 [39]

Mon	+ [40–42, 46, 47]	+ [43–47]	+ [41, 46, 47]	+ [47]	IFN-*γ* for PAR-1 [42]; thrombosis for PAR-1; APS for PAR-2 [46]	na

Mac	+ [41, 48, 50]	+ [41, 43, 48–51]	+ [41, 50]	+ [50]	LPS for PAR-1, -2, -3, and -4 [50]; dietary FA for PAR-2 [51]; MMIF for PAR-2 [77]; GM-CSF for PAR-1, -2, and -3 [41]; smoke for PAR-1 [48]	IL-4 for PAR-1, -2, and -3 [41]

TC	+ [52, 162]	+ [53, 162]	na	+ [162]	HIV for PAR-1 [52]	na

BC	+ [54]	+ [163]	na	na	na	na

DC	+ [55, 56]	+ [43, 57]	+ [56]	na	GC for PAR-2 [57]	LPS for PAR-1 and -3 [56]

MC: mast cell; Eos: eosinophil; Neu: neutrophil; Mon: monocyte; Mac: macrophage; TC: T cell; BC: B cell; DC: dendritic cell; RANTES: regulated upon activation normal T cell expressed and secreted; TNF: tumor necrosis factor; GM-CSF: granulocyte-macrophage colony-stimulating factor; IL: interleukin; IFN: interferon; APS: antiphospholipid syndrome; MMIF: macrophage migration inhibitory factor; LPS: lipopolysaccharides; FA: fatty acids; HIV: human immunodeficiency virus; AC: American cockroach; GC: German cockroach; na: not available.

**Table 2:** Expression of protease activated receptors (PARs) on structural cells.

Cell type	Expression of PARs				Regulator of PARs	
	PAR-1	PAR-2	PAR-3	PAR-4	Upregulation	Downregulation
EpC	+ [50, 101]	+ [50, 60, 101, 102]	+ [50, 101]	+ [50]	NE for PAR-2; LPS for PAR-1, -2, -3, and -4 [50]	na

EnC	+ [50, 62, 64–66, 100]	+ [50, 61, 64]	+ [50, 54, 66, 67]	+ [50, 66]	LPS for PAR-1, -2, -3, and -4 [50]; HCMV for PAR-1, -3, and -4 [66]; MMIF for PAR-1 and -2 [64]; TNF-*α*, IL-1*α*, and LPS for PAR-2 [63]	Phorbol ester for PAR-2 [63]; sheer stress for PAR-1 [62]

Fibro	+ [68–71, 164]	+ [53, 68–71, 164]	+ [68, 70, 71]	na	Malignancy for PAR-1 and -2 [164]; bFGF for PAR-2 [71]	PGE2 for PAR-2 [72]

SMC	+ [73, 75]	+ [74]	+ [75]	+ [75]	na	PGI2/PGE2 for PAR-1, -3, and -4 [75]

Kerat	+ [76, 82]	+ [11, 76, 82, 103, 104]	+ [76]	na	na	na

EpC: epithelial cell; EnC: endothelial cell; Fibro: fibroblast; SMC: smooth muscle cell; Kerat: keratinocyte; NE: neutrophil elastase; LPS: lipopolysaccharides; HCMV: human cytomegalovirus; MMIF: macrophage migration inhibitory factor; TNF: tumor necrosis factor; IL: interleukin; bFGF: basic fibroblast growth factor; PG: prostaglandin; na: not available.

**Table:** (a)

Agonist	Targeted cell	Response of cell
PAR-1		
Thrombin	PBMC [47]; A549 [92, 93]; Mon [94]; TC [95]; HPDF [96, 97]; CHAEC [98, 99]; EnC [54]	IL-1*β* [47], IL-6 [47, 94, 95], IL-8 [92, 96], MCP-1 [93], MMP-9 [97], MMP-12 [99], VCAM-1 [54], VEGF [98]
Trypsin	A549 [92, 93]; Mon [94]; TC [95]	Release of IL-8 [92]; MCP-1 [93]; IL-6 [94, 95]
SFLLR-NH_2_	A549 [58, 92, 93]; Mon [94]; HBEC [58]; TC [95]; CHAEC [98, 99]	IL-8 [92]; MCP-1 [93]; IL-6 [94, 95]; PGE2 [58]; VEGF [98]; MMP-12 [99]

PAR-2		
Tryptase	A549 [92, 93]; Neu [38]; Mon [94]; TC [95]; Astr [111]	Release of IL-8 [38, 92]; MCP-1 [93]; IL-6 [94, 95, 111]; LF [38];TNF-*α*, ROS [111]
Trypsin	A549 [92, 93]; Mon [94]; HPBF [68]; Eos [35]; MC [107]; HUVEC [108]	Release of IL-8 [68, 92, 108]; MCP-1 [93]; IL-6 [94]; G-CSF, VCAM-1 [68]; sys-LT, ROS [35]; tryptase [107]
Elastase	A549 [92, 93]; AEC [101]; Mon [94]	Release of IL-8 [92]; MCP-1 [93]; IL-6 [94]; MUC5AC [101]
GCE	MH-S [49]; RAW264.7 [49]	TNF-*α* [49]
WCE	PHKC [104]	Release of IL-8, GM-CSF [104]
SLIGKV-NH2	A549 [58, 92, 93]; HPBF [68]; HBEC [58]; TC [95]; HTEC [102]; Kerat [103]; Neu [38]; Eos [35, 36]; MC [107]; BEAS-2B, Calu-3 [109]	Release of IL-8 [38, 68, 92, 109]; MCP-1 [93]; PGE2 [58]; IL-6 [95, 109]; G-CSF [68]; VCAM-1 [68, 102]; GM-CSF [102, 109]; TNF-*α* [102]; MMP-1,10 [102]; TSLP [103]; LF [38]; sys-LT, ROS [35]; SOD [36]; His [107]
tc-LIGRLO-NH_2_	Neu [38]; MC [107]	Release of IL-8, LF [38]; His [107]

PAR-3		
Thrombin	A549 [92, 93]; Mon [94]; EnC [54]	Release of IL-8 [92]; MCP-1 [93]; IL-6 [94]; ICAM-1, VCAM-1 [54]

PAR-4		
Thrombin	A549 [92, 93]; Mon [94]	Release of IL-8 [92]; MCP-1 [93]; IL-6 [94]
GYPGQV-NH_2_	A549 [58, 92, 93]; Mon [94]; HBEC [58]	IL-8 [92]; MCP-1 [93]; IL-6 [94]; PGE2 [58]

**Table:** (b)

Antagonist	Targeted cell	Response of cell
PAR-1		
SCH 79797	HUVEC [165]; HDF [166]; MC [29]	Block thrombin induced CX3CL1 [165]; inhibit plasmin-induced IL-8, PGE [166]; inhibit trypsin and tryptase induced IL-4 release [29]
BMS 200261	PL [114]	Inhibit PAR-1 activation in response to thrombin [114];
RWJ-56110 [122, 167]; RWJ-58259 [123]	HSC [167]; HVC, PL [112, 113]	Reduced liver type I collagen [167], reduced liver calcium mobilization, and cellular functions (platelet aggregation and cell proliferation [112, 113])

PAR-2		
FSLLRY-NH2	HUVEC [108]; Astr [111]; MC [20, 29, 31, 32]	Inhibit trypsin-induced IL-8 [108]; alleviate IL-6 and TNF-*α* secretion [111]; inhibit trypsin and tryptase induced IL-4 release [20, 29, 31]; inhibit tryptase induced IL-13 release [32]
ENMD-1068	Eos [168]	Abolish tryptase-induced eosinophil recruitment [168]
GB88	HTEC [102]; HT29, A549, Panc-1, MKN1, MKN45, MDA-MB231, HUVEC [117]; Mac, MC [169]	Block PAR-2 agonist-induced increases in GM-CSF, IL-6, IL-8, and TNF-*α* [102]; ameliorate macrophage invasion and MC degranulation [169]; inhibit PAR-2 activated Ca(2+) release induced by trypsin or synthetic peptide and nonpeptide agonists [117]

PAR-4		
Pepducin P4pal-10	Neu [115]	Diminish neutrophil migration mediated by neutrophil expressed PAR-4 [115]

PBMC: peripheral blood mononuclear cells; A549: A549 epithelial cells; Mon: monocyte; TC: T cell; HPDF: human primary dermal fibroblast; CHAEC: cultured human airway epithelial cell; EnC: endothelial cell; HBEC: human bronchial epithelial cell; Neu: neutrophil; Astr: astrocytes; HPBF: human primary bronchial fibroblasts; Eos: eosinophil; MC: mast cell; HUVEC: human umbilical vein endothelial cell; AEC: airway epithelial cells; MH-S: alveolar macrophage cell lines; RAW264.7: peritoneal macrophage cell lines; PHKC: primary human keratinocyte; HTEC: kidney tubular epithelial cells; Kerat: keratinocyte; BEAS-2B: human airway epithelial cell line BEAS-2B; Calu-3: human airway epithelial cell line Calu-3; HDF: human dental pulp fibroblast-like cells; PL: platelet; HSC: hepatic stellate cell; HVC: human vascular cell; HT29: human colon adenocarcinoma cell line; Panc-1: pancreas epithelioid carcinoma line Panc-1; MKN1: human gastric carcinoma cell line MKN1; MKN45: human gastric carcinoma cell line MKN45; MDA-MB231: breast adenocarcinoma cell line MDA-MB231; Mac: macrophage; IL: interleukin; MCP-1: monocyte chemoattractant protein-1; MMP: matrix metalloproteinase; VCAM: vascular cell adhesion molecule; VEGF: vascular endothelial growth factor; PG: prostaglandin; LF: lactoferrin; TNF: tumor necrosis factor; ROS: reactive oxygen species; G-CSF: granulocyte colony-stimulating factor; cys-LT: cysteinyl leukotrienes; MUC5AC: mucin5AC; GM-CSF: granulocyte-macrophage colony-stimulating factor; TSLP: thymic stromal lymphopoietin; SOD: superoxide; His: histamine; ICAM: intercellular adhesion molecule.

**Table 4:** Roles of PARs in allergic diseases.

Disease	PAR involved	Response of cell or molecule
Rhinitis	PAR-1	VEGF secretion from CHAEC [98];
	PAR-2	Higher secretion rate and numbers of responding glands [122]; fluid hypersecretion of airway mucosa [123]; suppression of Cx26 production in human NEC [124]; IL-6 and IL-8 production in NECs [125]; tachykinin-mediated neurogenic inflammation [126];

Asthma	PAR-1	Bronchial inflammation is worsened [118]; expression of TGF-*β*1 to promote airway remodeling [119]; susceptible to AHR [120]
	PAR-2	Contractions of human airways [128]; bronchial SMC proliferation and migration [129]; increased anion secretion [130]; eosinophil infiltration, AHR, IgE levels to OVA sensitization [131] influx of eosinophils in BALF, protein leak in the bronchoalveolar space, increase BALF levels of the anaphylatoxins C3a and C5a [132]; increase IL-6 expression and induce the proliferation of asthmatic bronchial SMC [134]; augment TNF-*α*-induced MMP-9 expression [135]; generate Ca(2+) in HAECs [136, 138]; AHR, Th2, and Th17 cytokine release, serum IgE levels, and cellular infiltration [137]; CCL20 and GM-CSF production, increase recruitment and/or differentiation of mDC populations [139]; beta-adrenergic desensitization [140]; increase chemotactic activity for the HMC-1 mast cell line [141]; exaggerate cough [142]; chitinase-mediated [Ca(2+)] increase [13]; inhibit the development of airway eosinophilia and hyperresponsiveness in allergic mice through COX-2-mediated generation of the anti-inflammatory mediator PGE2 [143]; inhibit bronchoconstriction and airway hyperresponsiveness [144]

Skin disorders	PAR-2	Production of the TSLP and TNF-*α* [146]; modulation of the calcium ions in skin [147]; scratching behavior in mice [148, 149]; ear edema and infiltration of inflammatory cells [150]

Colitis	PAR-2	Inflammatory process in the intestinal mucosa [25]; impaired epithelial barrier [151]; somatic and visceral hyperalgesia and allodynia [152]; relaxation in colonic smooth muscle [153]; cytoskeleton contraction with subsequent changes in tight junction permeability [154]; visceral hypersensitivity [155]
	PAR-4	Contraction of the longitudinal muscle of colon [156]; antinociceptive [158]; increase paracellular permeability and myeloperoxidase activity [159, 160]; colonic hyposensitivity [157, 161]

VEGF: vascular endothelial growth factor; CHAEC: cultured human airway epithelial cell; Cx: connexin; NEC: nasal epithelial cell; TGF: transforming growth factor; AHR: airway hyperresponsiveness; SMC: smooth muscle cell; BALF: bronchoalveolar lavage fluid; TNF: tumor necrosis factor; HAEC: human airway epithelial cells; GM-CSF: granulocyte-macrophage colony-stimulating factor; mDC: myeloid DC; TSLP: thymic stromal lymphopoietin; CCL20: chemokine C-C motif ligand 20; PGE2: prostaglandin E2.
